# Advances in prenatal ultrasound diagnosis of fetal tympanic ring anomalies

**DOI:** 10.3389/fmed.2026.1706882

**Published:** 2026-03-13

**Authors:** Huixia Zhang, Ruibi Liao, Yuxin Li, Fang Huang, Liya Li, Guorong Lyu

**Affiliations:** 1 Department of Ultrasound, The Second Affiliated Hospital of Fujian Medical University, Quanzhou, China; 2 Department of Ultrasound, Anxi County Maternal and Child Health Hospital, Quanzhou, China

**Keywords:** developmental anomalies, fetus, genes, prenatal ultrasound, tympanic ring

## Abstract

The tympanic ring (TR) is an important component of the middle ear, and its developmental abnormalities can lead to serious consequences such as hearing loss. Prenatal identification of deep middle ear structural abnormalities, such as external auditory canal atresia, has long been challenging and has largely relied on indirect evaluation of auricular morphology. Recent advancements in ultrasound technology have enabled the prenatal detection of TR abnormalities and provided new imaging markers for earlier evaluation of external and middle ear development. This study aimed to evaluate the TR’s normal and aberrant ultrasonographic and other imaging manifestations, the important genes controlling its development, and associated syndromes. These aspects are summarized in this review, which also examines the limitations of the present research and its potential for clinical use. The findings demonstrate that ultrasonography of TR is a valuable additional screening technique for fetal external and middle ear abnormalities, especially when combined with microtia evaluation, which improves the ability to predict the risk of external auditory canal atresia. However, further research is required to standardize prenatal ultrasound diagnosis, establish measurement criteria, promote the technology, and integrate multimodal imaging into clinical diagnosis. This article aims to provide a comprehensive reference for prenatal diagnosis and genetic counseling.

## 1 Introduction

The first and second pharyngeal arches, together with the first pharyngeal cleft and the first pharyngeal pouch between them, form the developmental origins of both the outer and middle ears (1). Nodular mounds that arise from the mesenchyme between the pharyngeal arches fuse to produce the auricle during the fifth and sixth weeks of embryonic development. The tympanic ring, the descendant of the first pharyngeal arch, appears and ossifies around the ninth week. In addition to providing the tympanic membrane’s bony framework, the tympanic ring regulates the invagination of the external auditory canal, the development of tympanic membrane, and the positioning of malleus handle through epithelial–mesenchymal interactions (1). Structural defects may arise from disruption at any point in this developmental process. Thus, the normal development of the tympanic ring serves as a pivotal hub in the morphogenesis of the entire external and middle ear.

An important anatomical landmark in otologic surgery, the tympanic ring significantly influences surgical treatments including planning and execution (2). Precise anatomical measurements of fetal specimens have established normal reference values for the morphological features of the tympanic ring at various gestational weeks (3, 4). The use of tympanic ring as a screening marker was validated by later imaging studies. For a long period, prenatal ultrasound screening focused primarily on the auricular morphology. Assessing the patency and integrity of the middle ear structures and external auditory canal, deeply embedded in the temporal bone, remained a clinical challenge (5). Prenatal diagnosis of external auditory canal atresia or stenosis is challenging because direct inspection of the fetal external auditory canal is difficult. However, a study first reported in 2013 demonstrated that it was possible to clearly visualize and measure the fetal tympanic ring during mid-pregnancy using transvaginal or transabdominal ultrasound, thanks to advances in high-resolution ultrasound technology, specifically the application of three-dimensional (3D) ultrasound and multi-plane reconstruction techniques (6). This innovation provided a novel perspective for prenatal diagnosis by changing the assessment focus from the difficult-to-observe external auditory canal to the tympanic ring. Subsequent clinical studies further validated the clinical value of transbasal transverse ultrasonography in tympanic ring evaluation (7). Further the external auditory canal atresia diagnoses were highly consistent with aberrant tympanic ring findings in the cases included.

Thus, this article is aimed to thoroughly evaluate the most recent findings on the fetal tympanic ring’s genetic basis, imaging features, embryonic development, and association with congenital ear abnormalities. In order to provide an accurate theoretical foundation and useful guidance for prenatal diagnosis, genetic counseling, and early postnatal intervention, this article reviews the viability, diagnostic value, limitations, and future directions of using the tympanic ring as a novel marker for prenatal ultrasound screening of external and middle ear malformations by integrating anatomical, imaging, and molecular genetic evidence.

## 2 Methods

### 2.1 Literature retrieval and screening

The main objective of this article is to present a thorough account and analysis of the fetal tympanic ring’s development, prenatal imaging evaluation, associated genetic background, and clinical relationships. The publication references were retrieved after searching the PubMed, Web of Science, and China National Knowledge Infrastructure (CNKI) databases (till October 2025) in order to gather pertinent literature in a comprehensive manner. Medical subject headings (MeSH) terms and keywords associated with the review’s primary concepts were incorporated into the search. “Fetal tympanic ring,” “prenatal ultrasound,” “fetus,” “ear malformations,” “microtia,” and their synonyms were among the main search phrases. The specific PubMed search string included “tympanic ring” OR (“ear malformations” OR “congenital ear abnormalities” OR “microtia” OR “anotia” OR “microtia and atresia”) AND (“prenatal diagnosis” OR “prenatal”). Other datasets were searched using comparable techniques. To identify other pertinent research that might have been overlooked in the initial electronic search, reference lists of retrieved review articles and important publications were also manually searched.

Retrieved literature was imported into EndNote X9, and duplicates were removed. The study selection process was conducted based on the following predefined inclusion and exclusion criteria.

The inclusion criteria include: 1. Studies involving human fetuses or focusing on prenatal diagnosis. 2. Review articles, reputable textbooks, and original research articles (prospective or retrospective studies, case series, and case reports). 3. Research on the fetal tympanic ring’s embryology, anatomy, measurement parameters, imaging characteristics, and genetic correlations. 4. Articles written in either Chinese or English.

The exclusion criteria include: 1. Studies exclusively targeting postnatal or adult populations. 2. Studies lacking extractable relevant data. 3. Conference abstracts, unpublished manuscripts, or articles without full-text access.

### 2.2 Data extraction and quality assessment

Data extraction and literature screening were carried out independently by two researchers. Any disagreements were resolved through discussion or by consulting a third researcher. Authors, publication year, research design, sample size, gestational age range, assessment technique, primary measurement parameters, important findings, and details about abnormal tympanic ring and related genes or syndromes were among the data retrieved.

It should be particularly noted that this article is a narrative review and did not employ the quantitative quality assessment tools used in systematic reviews. This article indeed consolidates and reviews major research findings, clinical significance, and knowledge limits under each theme based on the existing literature.

## 3 Result

### 3.1 Growth and development of fetal tympanic ring

This section had seven research (3, 4, 6–10) that addressed the embryology, anatomy, and imaging measures of the fetal tympanic ring. Anatomical measures of fetal specimens and *in vivo* or specimen imaging measurements based on MRI or ultrasound are among the study techniques.

#### 3.1.1 Embryonic development

**Figure 1:**
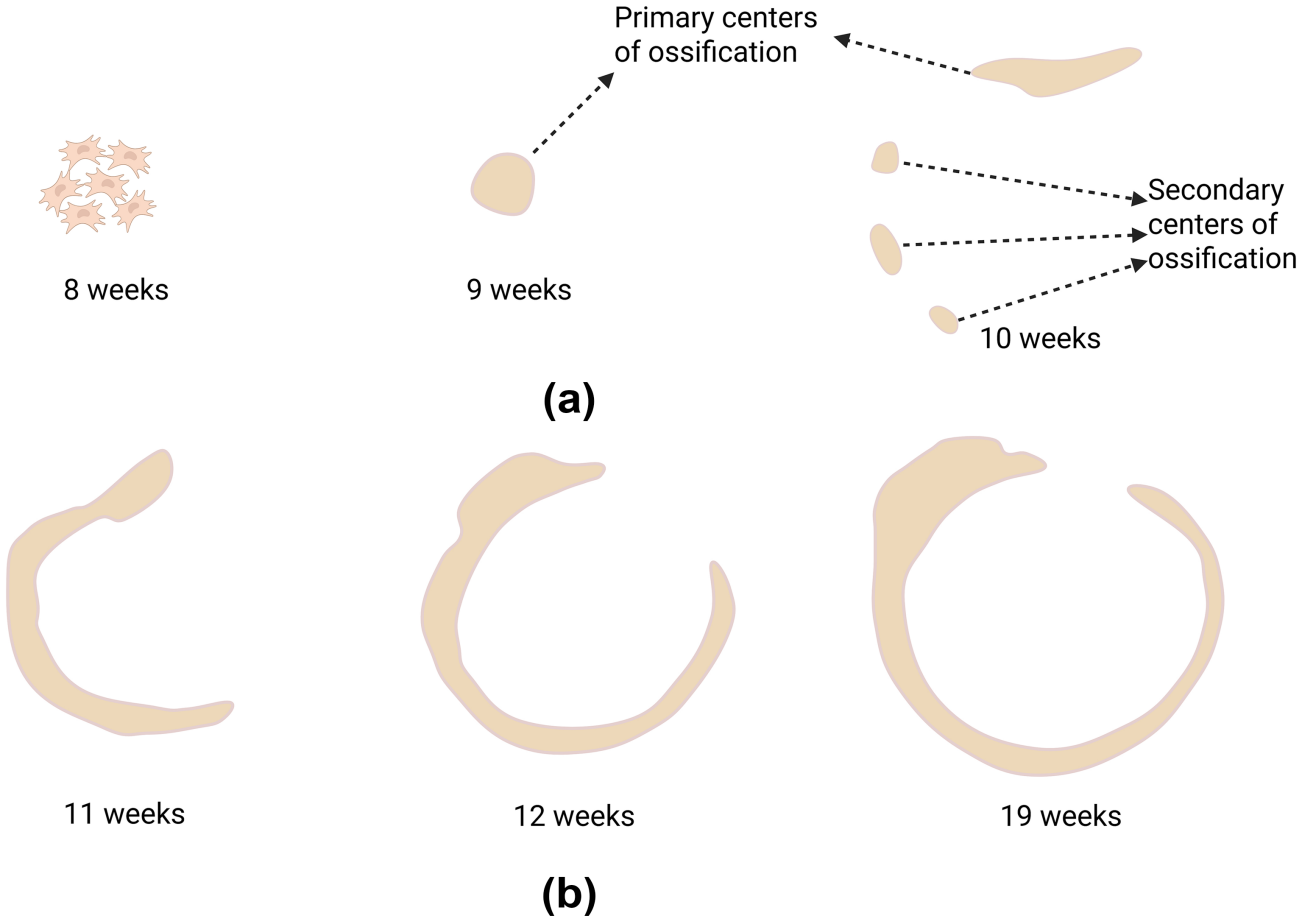
Embryonic development of the tympanic ring (104).

The primary source of the tympanic ring and other middle ear structures is Neural Crest Cells (NCCs), which migrate from the midbrain and hindbrain regions of the embryo into the first branchial arch (1, 11, 12). The tympanic ring initially appears as a region of mesenchymal cohesiveness beneath the first branchial arch (Meckel’s cartilage) during the early embryogenetic stages. At this stage, the tympanic ring has not yet developed a distinct ossification center. Near the bottom margin of the Meckel cartilage, the main ossification site of the tympanic ring, a small nodule of protochondrial tissue develops during the nineth week of embryonic development. The tympanic ring appears to develop several ossification centers after 10 weeks, revealing a C-shaped structure (Figure 1a). While several secondary ossification centers scattered throughout the tympanic ring’s body begin to emerge, the primary ossification center at the ring’s head is already established. The tympanic ring is situated between the first and second branchial arches at this time. The secondary ossification centers progressively unite to create a continuous C-shaped bone ring during the 11th week of embryonic development. The ossification process quickens and the tympanic ring’s diameter rises dramatically. The ossification process accelerates, and the tympanic ring’s greatly enlarges, and the diameter of the ring rises between the 12 and 16 weeks. The tympanic ring’s diameter and length continue to grow between 16 and 19 weeks of development, and the ossification process is nearly complete, creating a nearly complete ring structure (Figure 1b). The tympanic ring is almost completely formed by 23 weeks of embryonic development. The tympanic membrane’s three-layered structure is finished but not yet completely immersed in the ring’s sulcus tympanicus (8, 9). The tympanic ring develops to adult levels by 35 weeks (3). The tympanic ring eventually fuses with the surrounding structures to form the tympanic portion of the temporal bone during the late embryonic to birth phase. The anterior and posterior nodular bony processes develop toward the tympanic ring and integrate with one another throughout the first year of life. The external auditory canal is then formed by the tympanic ring expanding laterally to medially and cranially to caudally (13, 14). Around age five, the tympanic ring typically closes entirely (15). It should be emphasized that while modern imaging investigations have provided more insights into the *in vivo* development process, the comprehensive embryological timetable stated above is primarily based on traditional fetal anatomical studies. Nonetheless, sample gathering and observation techniques differ between the two.

#### 3.1.2 Growth and development characteristics

**Figure 2:**
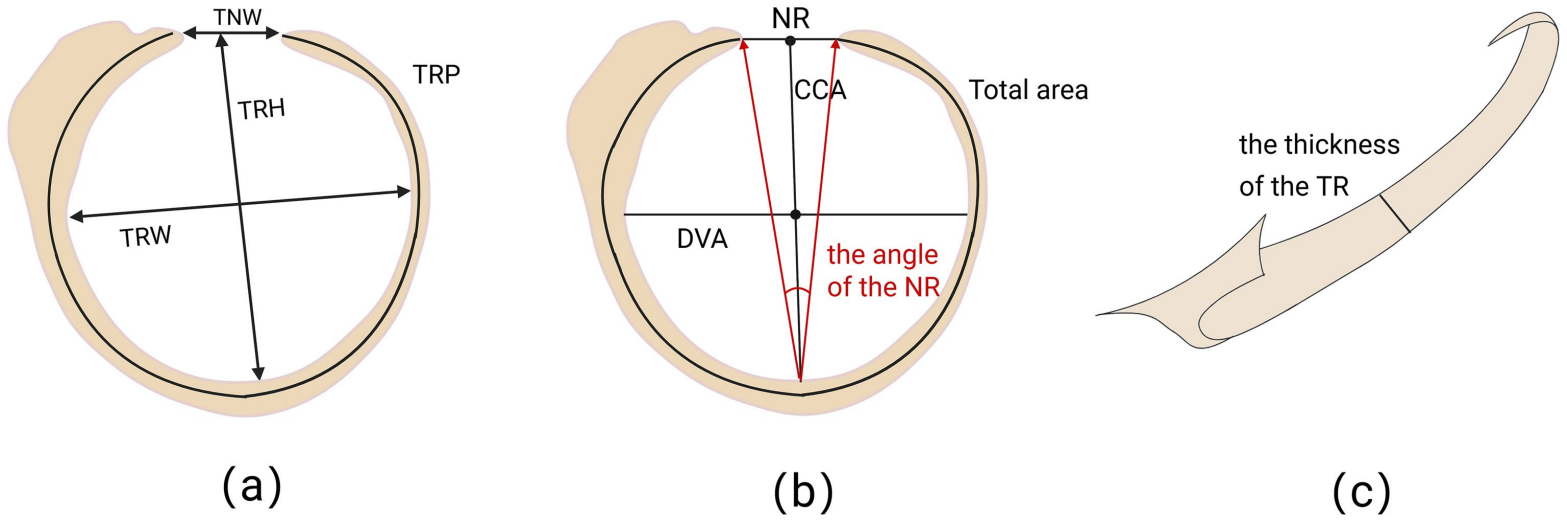
Anatomical parameters of the tympanic ring (TR). **(a)** TRH: Vertical diameter at the longest level of the TR; TRW: Horizontal diameter at the widest level of the TR; TNW: Width of the open portion of the TR; TRP: Perimeter of the TR. **(b)** NR: Length of the line connecting the two ends of the TR opening; CCA: Plumb line perpendicular to the direction of the inner surface of the TR through the midpoint of NR; DVA: Plumb line perpendicular to the direction of the inner surface of the TR through the midpoint of CCA. **(c)** Measurement of TR thickness by observation from above TR (105).

**Figure 3:**
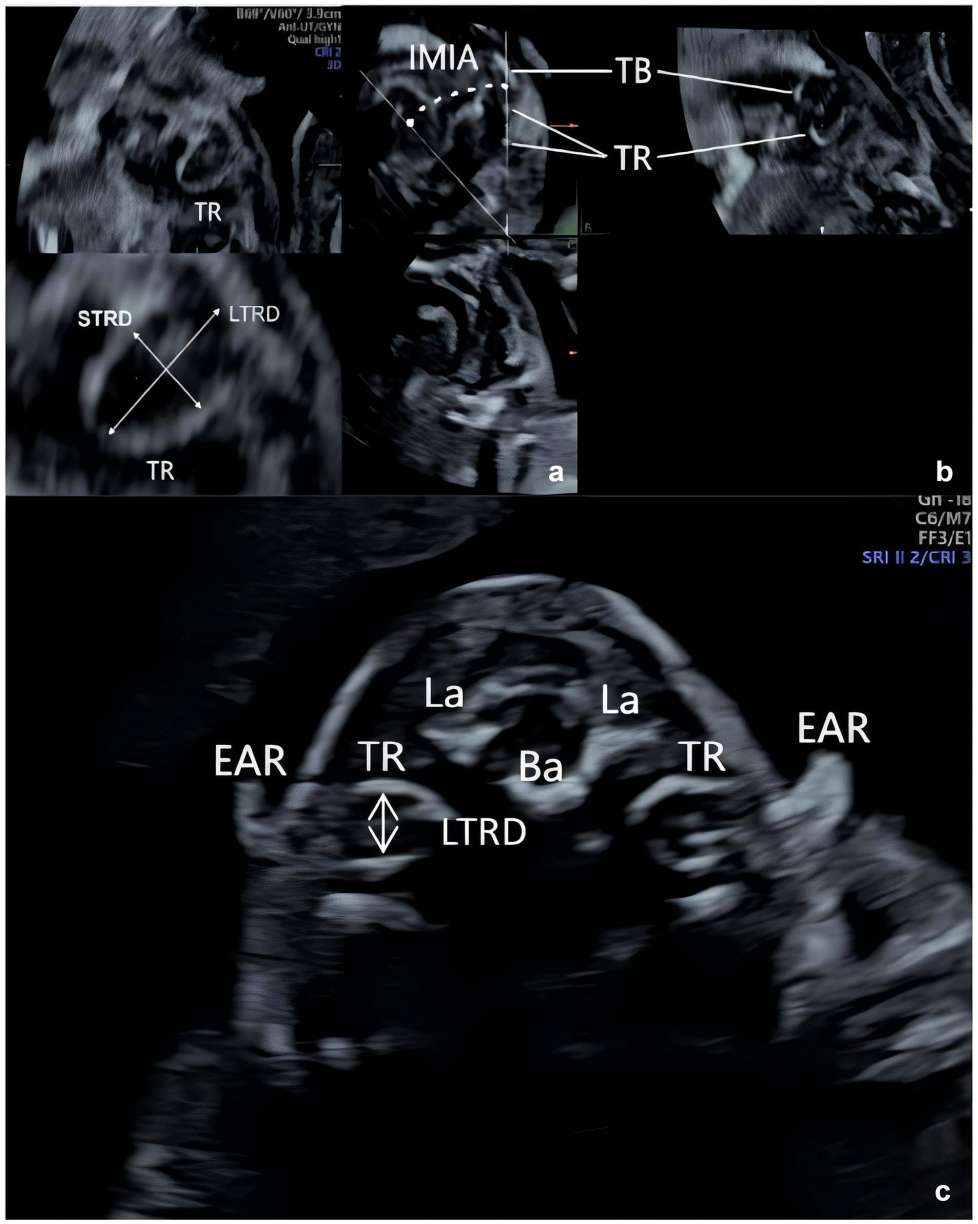
Schematic diagram of ultrasound measurements of the tympanic ring (image acquisition was performed following the method described in Wang Shanshan et al. (7) and Leibovitz et al. (6)). **(a)** LTRD: ongest diameter of the TR; STRD: shortest diameter of the TR. **(b)** IMIA: inferomedial inclination angle. **(c)** LTRD: anterior–posterior diameter of the TR.

Numerous research have provided information using anatomical or imaging techniques about the morphological measurements of the fetal tympanic ring (Table 1). Although the study designs and measuring techniques vary, they all show a variation pattern in the tympanic ring’s characteristics with increasing gestational age. The tympanic ring’s height, width, perimeter, and width of the notch of Rivinus all expand linearly between 20 and 30 weeks before stabilizing after the seventh month, according to a recent study that used direct anatomical assessment of fetal specimens (Figure 2a) (3). Although this study offers precise anatomical reference values, the findings may differ systematically from real ultrasound measurements because they are based on isolated specimens. The size of the tympanic ring increases linearly with gestational age within a certain period, according to the ultrasound technology used in live fetal investigations (Figure 3) (6, 7). These imaging studies lay the foundation for prenatal assessments. However, the sample size is very small, and there are currently no standardized ultrasound measurement protocols and diagnostic criteria, which limits the direct comparability of data between various studies. Another anatomical study shows that there was no significant difference in the size or shape of the tympanic ring between male and female embryos (Figures 2b, c) (4). These results assist doctors in in estimating the diameter of the tympanic ring during prenatal ultrasound imaging and detecting potential congenital abnormalities, particularly in the diagnosis of congenital hearing loss and developmental delays. The results also provide reference values for the normal development of the tympanic ring in the fetus. In addition, morphometric parameters of the tympanic ring can be used for preoperative evaluation of pediatric Transcanal Endoscopic Ear Surgery (TEES).

**Table 1:** Growth patterns of tympanic rings.

Literature	Research design and methods	Parameters	Growth pattern	Gestational week	Functional expressions (mm)
Leibovitz et al. (6) (Figures 3a,b)	Prospective observational study, transvaginal and transabdominal ultrasound measurement	Tympanic ring diameter (LTRD, STRD)	Diameter of the TR increases linearly with gestational week	12–32 weeks	Linear regression: LTRD, STRD and gestational week (*r* = 0.96)
		Angle of inclination (IMIA, AMIA)	Angle of inclination peaks in the second trimester		–
Beger et al. (3) (Figure 2a)	Anatomical measurement study, direct measurement of fetal specimens	TR height (TRH)	Linear increase, significant positive correlation with week of gestation	20–30 weeks	*y* = 1.328 + 0.281 × weeks (*p* < 0.001)
		TR width (TRW)			*y* = 1.284 + 0.258 × weeks (*p* < 0.001)
		TR perimeter (TRP)			*y* = 3.367 + 0.876 × weeks (*p* < 0.001)
		Width of the notch of Rivinus (TNW)			*y* = −0.603 + 0.188 × weeks (*p* < 0.001)
Nuñez-Castruita AND López-Serna (4) (Figures 2b, c)	Anatomical measurement study, morphological analysis of fetal specimens	Length of the cephalocaudal axes (CCA)	The length of CCA gradually increases and is positively correlated with week of gestation	12–37 weeks	–
		Length of the dorsoventral axes (DVA)	The length of DVA peaks at 21–24 weeks increases and then declines slightly		
		Total area	Total area peaks at 21–24 weeks increase and then declines slightly		
		Thickness at the level of the CCA	Gradual increase in thickness, positively correlated with week of gestation		
		Height at the level of the CCA	Gradual increase in height, positively correlated with week of gestation		
		Length of the notch of Rivinus (NR)	Slightly increased, peaked at 21–24 weeks then decreased slightly		
		The angle of the notch of Rivinus	The angle of the notch of Rivinus decreases gradually and is negatively correlated with gestational week		
Wang, Shanshan et al. (7) (Figure 3c)	Retrospective diagnostic study, prenatal ultrasound through the skull base cross section	Anterior–posterior diameter of the TR (LTRD)	The anteroposterior diameter of the TR is significantly and positively correlated with the week of gestation	12–31 weeks	*Y* = −0.15 + 0.28 × weeks (*r*^2^ = 0.775)
		The long diameter of the auricle	Anteroposterior diameters of the TR were significantly and positively correlated with the auricle length		*Y* = 2.32 + 0.21 x auricle length (*r*^2^ = 0.745)

LTRD, Longest tympanic ring diameter (front-back diameter); STRD, Shortest tympanic ring diameter; IMIA, Inferomedial inclination angle; AMIA, Anteromedial inclination angle; TRH, Vertical diameter at the longest level; TRW, Horizontal diameter at the widest level; TRP, TR perimeter; TNW, Width of the opening part of the TR at the level of the tympanic notch; CCA, Length of the cephalocaudal; DVA, Length of the dorsoventral; NR, Notch of Rivinus.

### 3.2 Imaging manifestations of fetal tympanic ring

**Figure 4:**
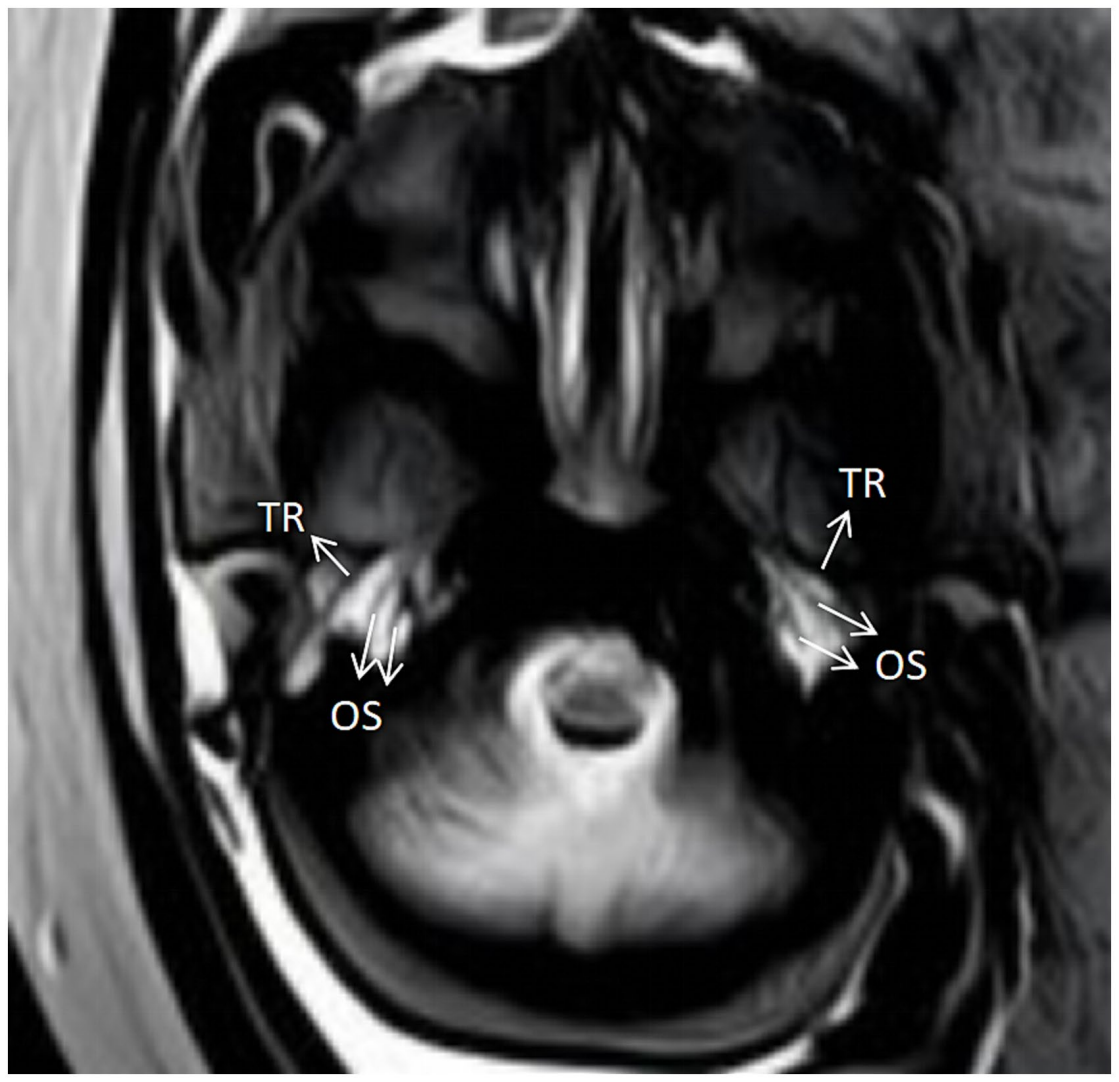
MRI manifestations of the tympanic ring (image acquisition was performed following the method described in Nemzek et al. (10)). TR: Tympanic ring; OS: ossicles.

The unossified tympanic ring makes it challenging to visualize the tympanic ring on ultrasound during the first trimester, and cranial ossification causes the rate of tympanic ring visualization to decline during the last trimester. Therefore, the second trimester (16–23 weeks) is considered the ideal period to evaluate the tympanic ring. A study first reported the ultrasound imaging of the fetal tympanic ring, which appeared on ultrasound images as a round or oval, thin-layered, strongly echogenic structure located below the squamous bone of the fetal skull near the posterior aspect of the temporomandibular joint (6). The tympanic ring was observed as a mirrored and symmetrical “C”-shaped structure when scanned in a transverse slice of the skull base by another study (7). The absence of the tympanic ring, its ambiguous appearance, or its anterior and posterior diameters that are noticeably smaller than the normal range indicate that the tympanic ring is underdeveloped or absent, which is typically accompanied by external ear malformations and atresia or stenosis of the external auditory canal. The fetal tympanic ring can also be demonstrated on other imaging, as seen in one article (10). As early as 19 weeks, the tympanic ring can be identified by computed tomography (CT) and magnetic resonance imaging (MRI). At that time, the ring appears as roughly nine-tenths of a circle, with a high-density osseous structure on CT scans. In the early stages, the ring is discontinuous, but in the later stages, it gradually fuses and shows up as a low-signal on the T2-weighted image of an MRI. The ring is situated near the auditory ossicles and is a horizontal structure (Figure 4). The tympanic ring is also seen as an ossified horizontal structure on the lateral and anteroposterior plain of the skull in weeks 22 and 23. Imaging of the tympanic ring is useful in assessing whether the fetal temporal bone is developing normally, and abnormalities in its morphology and ossification process may suggest congenital ear disease.

Prenatal ultrasound is highly reliable in diagnosing microtia and congenital defects that accompany it, and when combined with real-time 3D ultrasound can improve diagnostic accuracy. However, atresia of the external auditory canal is still tough to diagnose, according to domestic as well as international literature (5, 16–18). MRI has high soft tissue resolution and is used to assess structural abnormalities of the fetal ear, mainly through high-resolution T2-weighted sequences. Microtia or anotia may be suggested by the MRI showing an underdeveloped or absent auricle. For instance, a case diagnosed with an agnathia-otocephaly, which was demonstrated as an extremely low auricle and close to the midline on MRI (19). Postnatal neonatal appearance confirmed the imaging findings. MRI is considered as a more accurate way to determine external ear canal atresia than using the prenatal ultrasonography (20–22). A study reported a case of bilateral microtia with absence of external auditory canal in a trisomy 22 fetus at 36 gestational weeks by MRI, which was not detected by prenatal ultrasound (23). It is challenging to offer sufficient evidence before 25 weeks of gestation since the fetal external auditory canal cannot be seen until gestational weeks higher than 29 weeks (24). By displaying the signals of the tympanic cavity and the auditory ossicles (such as the hammer and anvil bones), MRI may be used to evaluate the middle ear for abnormalities. For example, fetuses with hypoplastic tympanic cavities have been found to have significantly reduced tympanic high signal (with amniotic fluid) in T2 sequences (25). In addition, MRI can be used to detect inner ear malformations (26). Despite the many advantages of MRI in the prenatal diagnosis of ear malformations, the examination is long, costly, and optimal after 25 weeks, when the development of most of the ear structures is almost at an end. Moreover the diagnosis of fetal anomalies can be devastating for the pregnant woman herself and for the family. Therefore, rather than being the primary option for prenatal diagnostics, MRI is frequently utilized as an adjunct to prenatal ultrasound. The delicate architecture of the embryonic ear can be easily seen on CT thanks to its high resolution. It is a crucial diagnostic tool for congenital atresia of the external auditory canal (27, 28). However, CT is difficult to use for prenatal diagnosis and contain ionizing radiation, making them unsuitable for routine examination of pregnant women and fetuses. Thus, an evaluation of the tympanic ring’s growth using ultrasound may help address the difficulty of diagnosing congenital hearing loss in utero and enable interdisciplinary counseling during the prenatal and postnatal stages.

### 3.3 Genes associated with fetal tympanic ring abnormalities

The development of the tympanic ring involves the regulation of several key genes and their signaling pathways (Table 2). The defects of the structure of the tympanic ring may result in the dysfunction of the middle and outer ear.

**Table 2:** Genes associated with tympanic ring development.

Gene name	Gene localization	Functionality	Related phenotypes or effects	Literature
Endothelin-1	6p24.1	Regulation of neural crest cells migration and differentiation	Absence leads to defective development of the tympanic ring and external auditory canal	Ivey et al. (29), Ozeki et al. (30), Clouthier et al. (31), Kanai et al. (32)
Gsc	14q32.13	Regulation of migration and early differentiation of neural crest cells	Deletion results in the tympanic ring being defective in the cell aggregation stage and completely absent	Rivera-Pérez et al. (36), Parry et al. (37), Anthwal et al. (38)
Prx	1q24.2		Mutations causing abnormal craniofacial development and ear shape	Rivera-Pérez et al. (33), Mallo et al. (34), Luquetti et al. (35)
Tbx1	22q11.21		Absence leads to shortening and thickening of the tympanic ring and developmental defects in other structures of the middle ear.	Moraes et al. (39), Xu et al. (40), Ankamreddy et al. (41)
Dlx5	7q21.3	Regulation of neural crest cell proliferation and differentiation	Absence resulting in mild abnormalities of the tympanic ring	Depew et al. (42), Gordon et al. (43), Kitazawa et al. (44)
Hoxa2	7p15.2	Regulation of the conversion of second branchial arch derivatives to first branchial arch derivatives	Absence leads to duplication of the tympanic ring and affects the formation of the external auditory canal	Alasti et al. (45), Minoux et al. (46), Kitazawa et al. (47)
Hoxa1/Hoxb1	7p15.2 / 17q21.32	Regulation of the development of the second branchial arch	Double mutation leads to degeneration of the second branchial arch and absence of the tympanic ring from the first branchial arch origin	Gavalas et al. (48)
Gas1	9q21.33	Involved in the regulation of the Hedgehog signaling pathway, which regulates the proliferation and differentiation of mesenchymal cells	Mutations leading to hypoplastic tympanic ring and absence of the external auditory canal.	Seppala et al. (49), Jia et al. (50), Xavier et al. (51)
Bapx1	4p15.32	Regulation of mesenchymal cell differentiation	Aberrant expression affects the anterior tympanic ring and hammer bone development.	Tucker et al. (52)
Tshz1	18q22.2		Loss of function leading to shortening and thickening of the tympanic ring, lateral displacement, and hammer bone deformity	Coré et al. (53)
Tcfap2a	6p24.3	Regulation of the development of the middle ear auditory ossicles and tympanic ring	Defects leading to the absence of the middle ear auditory ossicles and tympanic rings	Ahituv et al. (54), Feng et al. (55)
Runx2/Osx	6p21.1/12q13.13	Regulation of osteoblast differentiation and bone formation	Double heterozygous embryos presenting with hypoplastic or absent tympanic rings	Baek et al. (56)
COUP-TFII	15q26.2	Regulation of Sox9 expression and pre-osteoblast distribution	Absence leads to shortening and bifurcation of the tympanic ring, affecting the formation of the external auditory canal	Hsu et al. (57)
FGF10	5p12	Involved in FGF-FGFR signaling to regulate craniofacial skeletal development	Overexpression leads to craniofacial skeletal abnormalities such as the absence of tympanic rings.	Yoshioka et al. (58), Zhang et al. (59)

#### 3.3.1 Genes related to neural crest cell migration and differentiation

Tympanic ring growth originates primarily from neural crest cells, and endothelin-1 controls neural crest cell migration and differentiation by attaching to its receptor (Endothelin receptor type A, EDNRA). The tympanic ring and external auditory canal grow abnormally when endothelin-1 or its receptor is deleted (29–32). Neural crest cell migration and early differentiation are controlled by Goosecoid Homeobox (Gsc), Prx, and Tbx1. These genes are crucial for craniofacial development and coordinated ear shape, as demonstrated by mouse models with mutations in Gsc and Prx (33–35). The transcription factor Gsc serves as a downstream target for the endothelin pathway, which is expressed in the mesenchyme surrounding the first branchial cleft and regulates the number and distribution of precursor cells. Gsc deletion results in defective tympanic rings at the cell aggregation stage and complete deletion (36–38). Tbx1 mutant mouse models show that morphological abnormalities of the tympanic ring are associated with developmental defects in other structures of the middle ear, and that deletion of Tbx1 results in the shortening and thickening of the tympanic ring (39–41). Other genes that affect neural crest development include Dlx5 and Hoxa2. Dlx5 is a homeobox gene that is expressed in neural crest cells of the first branchial arch and regulates the proliferation and differentiation of these cells, and its deletion leads to mild abnormalities of the tympanic ring (42–44). Hoxa2 deletion leads to the conversion of the second branchial arch derivatives to the first branchial arch derivatives, resulting in tympanic ring duplication and affecting the formation of the external auditory canal (45–47). Double mutations in Hoxa1 and Hoxb1 lead to degeneration of the second branchial arch and deletion of the tympanic ring of the first branchial arch origin. Neural crest cells migrate from the neural tube to the first and second branchial arches, where they differentiate into mesenchymal cells that eventually form the tympanic ring (48).

#### 3.3.2 Downstream regulation and signal pathway genes

The Gas1 gene encodes a membrane-associated protein involved in the regulation of the Hedgehog signaling pathway, is expressed in mesenchymal cells surrounding the tympanic ring, and controls their proliferation and differentiation. Gas1 mutant mice exhibit hypoplasia of the tympanic ring and loss of the external auditory canal (49–51). Tucker et al. (52) found that Bapx1 expression partially overlapped with Gsc expression at E10.5, but Bapx1 predominantly regulated mesenchymal cell differentiation, affecting the development of the anterior part of the tympanic ring and the hammer bone. By the same mechanism as Bapx1, loss of Tshz1 function leads to shortening and thickening of the tympanic ring, lateral displacement, and malformation of the hammer bone (53). Null homozygote mice with deletion of the transcription factor Tcfap2a exhibit absence of middle ear auditory ossicles and tympanic rings (54, 55).

#### 3.3.3 Osteogenic differentiation and transcriptional regulation

Runt-related transcription factor 2 (Runx2) and Osterix/Sp7 transcription factor (Osx) affect tympanic ring development by regulating osteoblast differentiation and bone formation. Runx2 and Osx double heterozygous embryos exhibit underdeveloped or absent tympanic rings (56). Chicken ovalbumin upstream promoter–transcription factor (COUP–TFII) regulates Sox9 expression and anterior osteoblast distribution, and its deficiency leads to the shortening and bifurcation of the tympanic ring, which affects the formation of the external auditory canal (57). Excess fibroblast growth factor 10 (FGF10) may interfere with signaling of fibroblast growth factor–fibroblast growth factor receptor (FGF–FGFR), leading to craniofacial skeletal abnormalities in FGF10 transgenic mice, such as tympanic ring deletion (58, 59). Mutations or abnormal expression of these genes lead to defective tympanic ring development, which in turn affects the normal function of the middle and outer ear.

#### 3.3.4 Clinical relevance and syndromes

The connection between pertinent gene mutations and anomalies in the human ear development has been further demonstrated by multiple clinical investigations. A research reported that four unrelated patients with the syndrome mandibulofacial dysostosis with alopecia (MFDA) all harbored *de novo* missense variants in EDNRA (60). In affected siblings with Auriculocondylar syndrome (ACS) from a consanguineous family, a homozygous substitution at the prion protein cleavage site of the EDN1 precursor protein was identified. Termination mutations in EDN1 and missense mutations in conserved residues of the mature peptide segment were discovered in two vertically inherited families with question mark ears (61). Using information from 1,459 patients, a review also summarized the relationship between microtia and genes such as Treacle ribosome biogenesis factor 1 (TCOF1), SIX homeobox 2 (SIX2), Homeobox A2 (HOXA2), and GSC (62). A recessive PRRX1 mutation was identified in a newborn with cleft ear deformity born to consanguineous parents, inherited from phenotypically normal parents (63). TBX1 is a gene hemizygously deleted in 22q11DS patients. Most individuals with 22q11.2 deletion syndrome (22q11DS) exhibit middle and outer ear abnormalities, with some also presenting inner ear malformations (64). A report described a three-generation family with hearing loss and craniofacial defects due to a deletion in the DLX5/DLX6 adjacent enhancer region and chromosomal inversion (65). A homozygous missense mutation was identified in Homeobox B1 (HOXB1) across two families, with phenotypes including bilateral facial paralysis, hearing loss, and strabismus (66). No Bagpipe homeobox homolog 1/NKX3-2 (BAPX1) mutations were detected in 105 patients with oculo–auricular–vertebral spectrum (OAVS), but its epigenetic dysregulation plays a significant role in OAVS (67). A contribution identified Teashirt zinc finger homeobox 1 (TSHZ1) deletions in four patients with congenital atresia of the ear (CAA) from two families and characterized two TSHZ1 mutations in 11 patients with non-syndromic bilateral CAA (68). Mutations in TFAP2A frequently cause branchial–ocular–facial syndrome (BOFS), a rare autosomal dominant disorder (69). Cleidocranial dysplasia (CCD), an autosomal dominant skeletal dysplasia, results from inactivating mutations in the CBFA1/RUNX2 gene and is often associated with hearing loss (70). However, hearing loss primarily results from conductive middle ear issues secondary to ossicular structural abnormalities (71). A retrospective study examined clinical features in 17 individuals with heterozygous NR2F2 (COUP-TFII) variants, including congenital and acquired microcephaly, facial dysmorphism, and hearing loss (72). Mutations in the FGF10 gene cause lacrimo–auriculo–dento–digital (LADD) syndrome, characterized by involvement of the lacrimal and salivary systems, cup-shaped ears, hearing loss, and dental abnormalities (73). It should be noted that developmental abnormalities or dysfunctions of the inner ear mainly lead to congenital deafness. Studies have shown that about 60% or more of the etiology of deafness is related to genetic factors, and the most common deafness genes include Gap junction protein beta 2/connexin 26 (GJB2), Solute carrier family 26 member 4/pendrin (SLC26A4), Mitochondrially encoded 12S rRNA (MT-RNR1), and Gap junction protein beta 3/connexin 31 (GJB3) (74).

### 3.4 Diseases associated with fetal middle ear malformations

A number of otologic problems can arise from improper development or lesions of the tympanic ring, which is crucial to the development of the ear (75). In this article, the major diseases and syndromes associated with fetal middle ear malformations is described.

#### 3.4.1 Common syndromes

Malformations or syndromes associated with the middle ear usually manifest as stenosis or atresia of the external auditory canal and malformations of the auditory ossicles, leading to conductive hearing loss (Table 3). Particularly in patients with syndromes, these abnormalities frequently coexist with abnormalities of other systems (such as the heart, kidneys, or spine). Common syndromes include Treacher Collins Syndrome (TCS), Branchio–oto–renal Syndrome, Goldenhar Syndrome, and CHARGE Syndrome (76, 77). TCS is an autosomal dominant craniofacial dysplasia caused by mutations in the TCOF1 (>90%), POLR1D, or POLR1C genes, resulting in abnormal development of the first and second branchial arches, with the core features of bilaterally symmetrical outer and middle ear and maxillofacial malformations (78–80). Hemifacial microsomia (HFM) is a congenital craniofacial malformation caused by anomalous development of the first and second branchial arches, which may present as unilateral or bilateral anomalies of varying severity, and which may also be recognized as Goldenhar syndrome or OAVS, a group of disorders that are etiologically heterogeneous (81–84). Oculoauriculofrontonasal Syndrome (OAFNS) is a rare craniofacial–ear malformation syndrome with core manifestations of the overlapping features of Frontonasal Dysplasia (FND) and OAVS, of unspecified etiology, which may be associated with abnormal development of the branchial arches (85–87). CHARGE syndrome is a multisystem congenital anomaly syndrome caused by mutations in the CHD7 gene (>90%), which belongs to the neural crest cells dysplasia disorders, and when suspected prenatally, posterior nostril atresia should be prioritized to avoid endangering neonatal respiration (88). Noonan syndrome (NS) is an autosomal dominant multisystem disorder of the RAS–MAPK signaling pathway, typically characterized by peculiar facial features, short stature, congenital heart disease, and hearing abnormalities caused by mutations in PTPN11, SOS1, RAF1, and SHOC2 (89, 90). Waardenburg syndrome (WS) is a group of autosomal dominant disorders with abnormalities of neural crest cell development, characterized by a peculiar facial appearance and congenital sensorineural hearing loss. Although combined middle ear malformations are rare, the possibility of mixed hearing loss should be guarded against (91). Townes–Brocks syndrome (TBS) is an autosomal dominant disorder caused by heterozygous mutations in the SALL1 gene, with the classic triad of anorectal malformations (e.g., anal atresia), thumb malformations (e.g., tripartite thumb, or radial polydactyly), and ear malformations (92). Multiple Synostoses Syndrome Type 1 (SYNS1) is a syndrome characterized by multiple fused joints, hearing loss, and a peculiar facial appearance, inherited as an autosomal dominant trait, mainly caused by mutations or deletions in the NOG gene (93–95).

**Table 3:** Malformations or syndromes associated with the middle ear.

Disease type/syndrome	Disease spectrum (clinical manifestations)
Treacher Collins syndrome	Malformations of the outer and middle ear, commonly with stenosis or atresia of the external auditory canal; Auditory ossicle deformity, resulting in conductive hearing loss; Facial hypoplasia, hypoplastic mandible; May be accompanied by inner ear malformations
Branchio-oto-renal syndrome	Malformations of the outer and middle ear, commonly with stenosis or atresia of the external auditory canal; Auditory ossicle deformity, resulting in conductive hearing loss; Kidney malformations, which may be associated with abnormal kidney function; Fistulae or cysts in the neck
Goldenhar syndrome/OAVS/HFM	Malformations of the outer and middle ear, commonly with stenosis or atresia of the external auditory canal; Auditory ossicle deformity, resulting in conductive hearing loss; Facial hypoplasia, hypoplastic mandible; Spinal deformities, which may be associated with vision problems
Oculoauriculofrontonasal Syndrome	Frontonasal Dysplasia, ocular hypertelorism, wide nasal bridge, facial cleft; Ear deformities, preauricular neoplasm, microtia, abnormal ear position; epibulbar dermoids, blepharocoloboma, microphthalmia/anophthalmia, cataracts; Mandibular hypoplasia, mandibular fissures, spinal anomalies, thyroid deficiency, high incidence of nasal polyps
CHARGE syndrome	Malformations of the outer and middle ear, commonly with stenosis or atresia of the external auditory canal; Auditory ossicle deformity, resulting in conductive hearing loss; Inner ear malformations, such as cochlear hypoplasia; Cardiac malformations, ocular malformations, posterior nasal aperture atresia, etc.
Noonan syndrome	External ear deformities, low-set ears, auricular supination, bat ear, thickened helix, dystrophic calcification of the auricle; Hearing loss; Otitis media with effusion, auditory ossicles deformity
Jervell and Lange-Nielsen syndrome	Congenital deafness, usually sensorineural hearing loss; ECG showing long QT intervals, which may lead to arrhythmias; No middle ear malformation, but may be associated with other heart problems
Waardenburg syndrome	Malformations of the outer and middle ear, commonly with stenosis or atresia of the external auditory canal; Auditory ossicle deformity, resulting in conductive hearing loss; Skin and hair pigmentation abnormalities, such as albinism; May be accompanied by inner ear malformations
Nager syndrome	Malformations of the outer and middle ear, commonly with stenosis or atresia of the external auditory canal; Auditory ossicle deformity, resulting in conductive hearing loss; Facial hypoplasia, hypoplastic mandible; May be accompanied by inner ear malformations
Townes-Brocks syndrome	Malformations of the outer and middle ear, commonly with stenosis or atresia of the external auditory canal; Auditory ossicle deformity, resulting in conductive hearing loss; Renal malformations, which may be associated with abnormal kidney function; Hedratresia or rectal malformations
VACTERL syndrome	Malformations of the outer and middle ear, commonly with stenosis or atresia of the external auditory canal; Auditory ossicle deformity, resulting in conductive hearing loss; Spinal deformities, hedratresia, cardiac malformations, tracheoesophageal fistulae
Multiple Synostoses Syndrome Type 1	Stirrup fixation or auditory ossicle deformity, resulting in conductive hearing loss; Skeletal abnormalities, proximal symphalangism, brachydactylia or incomplete syndactyly; Facial deformity, semicylindrical nose, short philtrum, hypoplastic wings of nose; Eye abnormalities, possibly with amblyopia or strabismus
Agnathia-Otocephaly Complex	Abnormal ear position or malformation of the middle ear, which may be accompanied by auditory ossicle deformity; Complete agnathus or severe micrognathia; Microstomia, tongue agenesis or hypoplasia
Scalp-Ear-Nipple syndrome	Malformations of the outer ear, such as microtia, cup ear, and excessive folding of helix; Congenital hypoplasia of the scalp, glabrous nodules at the back of the head; Absent or underdeveloped nipples,micromazia; Finger (toe) deformities, such as 2-3 syndactyly; Mild atopic dermatitis, abnormal facial features such as thinning hair, peculiar facial features
Cryptotia	The upper part of the auricle (anthelix, fossae triangularis auriculae) is covered with skin, forming a "pocket-like" structure; Loss of the temporoauricular sulcus, adhesion of the auricle to the temporal skin; Shortening of the crus of helix, flattening of the upper contour of the auricle; May be accompanied by mild stenosis of the external auditory canal

#### 3.4.2 Other important syndromes and deformities

In addition, some specific malformation syndromes are of specific concern. The Agnathia–Otocephaly Complex (AOC) is a rare and usually fatal congenital malformation caused by abnormal development of the first branchial arch, characterized by a hypoplastic or absent mandible (agnathia/micrognathia), ear malformations, microstomia, and hypoplasia of the tongue, with an abnormal ear position suggesting that the external auditory canal and the structures of the middle ear may be displaced or hypoplasia (96). A study reported two cases of Cyclopia, an extremely severe form of Holoprosencephaly (HPE) caused by failure of embryonic forebrain splitting, which manifests as a monocular eye and features of AOC, and which may be combined with inner ear malformations that result in sensorineural deafness (97). Scalp–ear–nipple syndrome (SENS) is a rare autosomal dominant disorder caused by a mutation in potassium channel tetramerization domain containing 1 (KCTD1), resulting in a multisystemic malformation syndrome characterized by scalp defects, ear malformations, and nipple defects. This disorder may be associated with a mutation in the gene that results in a loss of the ability to bind to the transcription factor AP-2α, which affects the development of the craniofacial and ectodermal layers and interferes with the Wnt/*β*-catenin pathway, affecting cell proliferation (98–100). Cryptotia is a congenital auricular malformation characterized by the upper one-third of the auricle being partially embedded under the temporal skin, resulting in the absence of the temporoauricular sulcus, and is usually an isolated deformity, with a small number of comorbidities with other ear malformations, such as microtia. However, it is necessary to be vigilant about the possibility of the concurrent occurrence of other syndromes (101, 102). Ultrasound is a noninvasive test that can indicate the fetal ear disease risks, but the prenatal detection rate of middle ear malformations alone is low and should be combined with other indicators. Prenatal confirmation of the syndrome still relies on both the genetic testing and phenotypic association analysis.

## 4 Discussion

This method converts the prenatal assessment of middle ear structures from indirect inference to direct observation by changing the assessment goal from the “unobservable external auditory canal” to the “visualizable bony tympanic ring” (6, 7, 103). According to available data, aberrant tympanic ring presentations are highly consistent with external auditory canal atresia, particularly when assessed in combination with microtia, thereby greatly improving the predictive power for external auditory canal atresia. However, before this technology can be applied in clinical settings, a significant amount of work remains.

### 4.1 An urgent need to set diagnostic thresholds and technical standards

The reliability and generalizability of the present statistical results are limited, since classifications of “normal” and “abnormal” tympanic ring are primarily based on small-sample retrospective studies without large-scale sample validation. Additionally, there is no single ultrasonic measuring standard or diagnostic threshold for the tympanic ring, and differences in measurement techniques across studies making diagnosis inconsistent and difficult to compare. In addition, several factors may explain why “the tympanic ring not being displayed” occurs. While structural deficiency may be one of the cause, technical issues such as incorrect fetal positioning or unsuitable gestational age may also be accountable.

### 4.2 Fundamental drawbacks of the existing evidence framework

The following flaws remain in the current evidence system in addition to the previously noted technical standards problems. First off, there is not a large-scale prospective cohort study to confirm that measuring the tympanic ring is a reliable diagnostic method. Second, there is little correlation between genes and ultrasonography phenotypes, and the corresponding pattern has not yet been established. Lastly, prognostic information is lacking. It is challenging to evaluate the long-term benefits of this approach because there are few publications on the postnatal hearing results, surgical consequences, and long-term quality of life of fetuses with aberrant tympanic rings detected prenatally.

### 4.3 Cooperative multimodal imaging positioning and stratification techniques

The main screening method is ultrasound, which is appropriate for systematic screening throughout the second trimester due to its benefits, which include broad accessibility, real-time capacity, and a radiation-free nature. When ultrasound results are not conclusive, MRI is the secondary verification method that can provide direct imaging of both the external auditory canal and of the development of the ossicles and tympanic chamber. With its ability to provide a thorough evaluation of the bony anatomy for surgical planning, CT remains the gold standard for postpartum imaging. Because CT is only performed after delivery, fetuses with aberrant tympanic rings or microtia discovered by ultrasound should be referred to a prenatal diagnosis center for evaluation by qualified radiologists to determine whether fetal MRI is required. By avoiding over-medicalization, this stratification approach can increase diagnostic precision and guide future, more focused tests and consultations.

### 4.4 Multidisciplinary management’s clinical value and significance

Prenatal tympanic ring anomality detection is crucial not only for diagnosis but also for warning and preparation. In addition to offering clear guidelines for targeted genetic testing that will support genetic counseling and prenatal diagnosis, this study should prompt systematic structural screening by the obstetrics and ultrasound departments to prevent missed diagnoses of related heart, kidney, spine, and facial malformations. Newborn hearing screening and auditory brainstem response testing are examples of interventions that audiology and hearing clinics can schedule in advance for postnatal hearing assessment and intervention windows. Early knowledge of the many options for hearing restoration can greatly ease postpartum stress and decision-making for families. Emphasizing that the ultrasonic evaluation of the tympanic ring should be explicitly characterized as a collaborative assessment indicator, rather than an independent diagnostic basis, is especially crucial. Any decision to end a pregnancy should not be based on the ultrasound finding of a single anomaly in the tympanic ring.

### 4.5 Future directions

In future, a multifaceted cooperative effort may be needed for the clinical application of prenatal ultrasonography examination of the tympanic ring. Large-scale, prospective, multicenter studies are anticipated to establish normal reference ranges for tympanic ring measurement parameters across different populations and gestational weeks, while defining standardized measurement methods. The potential of new technologies, such as high-frequency probes and 3D ultrasound, to improve the image quality and measurement accuracy needs more investigation. A strong biological basis for this imaging biomarker may be provided by mechanistically clarifying the causative link between tympanic ring developmental anomalies and ear deformities, through animal model research. For precision medicine to advance, more genes appropriate for prenatal diagnosis should be investigated and validated in future. Integrating genetic testing with the clinical pathways is already essential for guiding individualized interventions, improving patient outcomes, and providing genetic counseling.

Additionally, there is a great deal of therapeutic promise for the use of artificial intelligence (AI) technologies, especially deep learning, in the analysis of fetal tympanic rings using ultrasound images. AI models may efficiently and objectively locate tympanic rings through automated identification and segmentation, enabling precise morphological measurements. This has the potential to turn screening from accidental discoveries into a routine procedure. Building imaging-based “genotype–phenotype” association prediction models also enables effective prescription of specific genetic testing procedures and prioritization of high-risk genetic disorders. Further, collaborative efforts across multiple centers are needed to build richer annotated datasets and develop more interpretable AI models, thereby facilitating their effective transition from research tools to routine clinical practice.

Finally, prenatal ultrasound assessment of the fetal tympanic ring represents a promising technology that offers a novel window into fetal external and middle ear development. Although challenges remain in terms of technical stability and diagnostic consistency of the assessment method, continuous research and collaborative innovation are essential for its successful clinical translation.

## 5 Conclusion

In finalization, evaluating the fetal tympanic ring using ultrasound is a promising imaging biomarker for the prenatal identification of middle-ear abnormalities. Examining ossicle developmental patterns using ultrasound also presents a significant prospect for accurate diagnosis in the future. The primary benefit is that a systematic tympanic ring evaluation can help predict the likelihood of concurrent external auditory canal atresia when a small ear deformity is found. This serves to direct more thorough fetal structural screening and subsequent targeted examinations (such as fetal MRI). However, there are still issues with the present use of this technology in regular screening, such as the dependence on the operator’s experience, the variety of reasons for the “no display” phenomenon, and the absence of consistent measurement standards and diagnostic thresholds. Consequently, rather than serving as a stand-alone diagnostic foundation, the tympanic ring evaluation should be viewed as a crucial collaborative assessment tool. In order to eventually reach widespread application in precise prenatal diagnosis and stratified management, future research should concentrate on developing standardized tympanic ring evaluation methods, elucidating its diagnostic efficacy, and investigating associations with genetic indicators.
